# Evaluating the Role of First Polar Body Morphology on Rates of Fertilization and Embryo Development in ICSI Cycles

**Published:** 2011-09-23

**Authors:** Iman Halvaei, Mohammad Ali Khalili, Mehrdad Soleimani, Mohammad Hossein Razi

**Affiliations:** Research and Clinical Center for Infertility, Shahid Sadoughi University of Medical Sciences, Yazd, Iran

**Keywords:** First Polar Body, Oocyte Morphology, ICSI, Fertilization Rate, Embryo Development

## Abstract

**Background:**

Recent studies have demonstrated that morphology of the first polar body (1^st^PB) is related to oocyte viability, which can be used as a prognostic tool to predict oocyte performance and pregnancy outcomes in an intracytoplasmic sperm injection (ICSI) program. According to some studies, there is a correlation between oocyte performance and 1^st^PB morphology, while others have not reported any correlation. The objective of this study is to evaluate the role of 1^st^PB morphology on rates of fertilization and embryo development in ICSI cases.

**Materials and Methods:**

In this prospective study morphological characteristics of 470 metaphase II (MII) oocytes were assessed in 80 ICSI cycles. The women were ages 21-42 years (mean 32.6 ± 0.2). Their oocytes were retrieved after a hyperstimulation protocol. After denudation, all oocytes were evaluated for 1^st^PB morphology. The oocytes were divided into two groups of A (normal 1^st^

**Results:**

Twenty-seven percent of oocytes had fragmented 1^st^PB, while the remainder was associated with other morphological abnormalities. A total of 46.1% and 26.9% of oocytes showed double and multiple defects, respectively. RF was the most common abnormality observed in group B. No significant differences in women’s’ ages between groups A and B were noted (p=0.3). A total of 179 and 107 oocytes (61.5% vs. 59.8%) were fertilized in groups A and B, respectively (p=0.7). The rates of good embryo formation for A and B groups were 66.5% and 55.6% (p=0.07), and cleavage rates were 77.7% and 68.5%, respectively (p=0.09).

**Conclusion:**

The data demonstrated that 1^st^PB morphology does not appear to be a prognostic factor for rates of fertilization and embryo development in ICSI cycles.

## Introduction

One of the most important factors that determine success in assisted reproductive technology (ART) is the oocyte. It is clear that the quality of oocytes can affect fertilization and embryo development (1). By introduction of intracytoplasmic sperm injection (ICSI), many couples with male factor infertility have taken this opportunity to overcome their infertility. One of the capabilities of ICSI is the evaluation of oocyte morphology and maturity after denudation of cumulus cells for microinjection. The majority of all metaphase II (MII) oocytes (60%-70%) have at least one morphological abnormality (2). Many studies have reported the effect of morphological characteristics of oocytes on fertilization rate and embryo development. The outcome of ART is dependent on both patient parameters and embryo variables (3). Evaluation of first polar body (1^st^ PB) morphology is useful for distinguishing the post-ovulatory age of the oocyte (4). Correlation between oocyte morphology and ICSI outcome is still a matter of controversy (5-9).

Ebner et al. (10) have reported that the 1^st^ PB shape can affect fertilization rate and embryo quality in ICSI cycles. Recent studies have also demonstrated the relationship of 1^st^ PB morphology to mature oocyte viability, which may be used as a prognostic factor to predict oocyte performance and pregnancy achievement after an ICSI treatment (11, 12). Some studies have shown a correlation between oocyte performance and 1^st^ PB morphology during ICSI treatment cycles (9,10,13-15). However, others did not show any correlation between 1^st^ PB and ICSI outcomes (16-19).

Additionally, the correlation between blastocyst formation, implantation rate and 1^st^ PB morphology has been reported by Ebner and colleagues in 2002 (14). Germinal vesicle breakdown (GVBD) and simultaneous extrusion of the 1^st^ PB shows completion of the first meiotic division in human oocytes. As a result, the 1^st^ PB is a marker which indicates that the oocyte is ready to undergo the fertilization process. This event is synchronized with nuclear and cytoplasmic maturation (20) The main goal of this prospective study is to evaluate the correlation of 1^st^ PB morphological characteristics with rates of fertilization and embryo development in ICSI cycles.

## Materials and Methods

### Patient selection

In this prospective study, we evaluated morphological characteristics of 470 MII oocytes from 80 ICSI cycles. Maternal age was between 21-42 years. All patients underwent ICSI treatment at Yazd Research and Clinical Center for Infertility between April 2010 and August 2010. This study was approved by our Center’s Ethics Committee. Patients signed informed consents.

### Controlled ovarian hyperstimulation

In most patients, controlled ovarian hyperstimulation was undertaken with GnRH agonist downregulation, followed by rec FSH. An antagonist protocol was also used. Next, 10,000 IU of human chorionic gonadotrophin (hCG, i.m. DRG Co., Germany) was administered. The ovarian response was controlled by transvaginal ultrasound and serum estradiol concentration. Oocyte retrieval was done approximately 36 hours after hCG injection under transvaginal ultrasound-guidance.

### Semen analysis and sperm preparation

Semen analysis was done according to a WHO laboratory manual (21). Sperm specimens were obtained by ejaculation or testicular biopsy in azoospermic patients. We used a Makler chamber and light microscopy at ×200 magnification to determine sperm counts and motility evaluation. Progressive and nonprogressive spermatozoa were reported as percentages. Sperm morphology was evaluated using Giemsa staining. All sperm preparations were performed using the swim-up or density gradient techniques (22). For swim-up, 1 ml of semen was mixed with 3 ml of Ham’s F10 medium (Seromed Co., Germany) supplemented with 10% human serum albumin (HSA). After gentle mixing, the sample was centrifuged twice (2000 rpm for 10 minutes, followed by 5 minutes). Following the removal of the supernatant, 0.2-1.0 ml of the culture medium was added, dependent upon the size of the pellet and the quality of the original sample. The suspension was then incubated at 37°C in 5% CO_2_ until use.

### ICSI procedure

After oocyte aspiration, the oocytes were incubated for about 4 hours, then denudation from cumulus cells occurred with the use of 80 IU hyaluronidase/ ml (Sigma Chemical Co., USA) along with the mechanical aid of appropriate Pasture pipettes. Each of the MII oocytes were washed in culture media and before microinjection, their morphological characteristics were evaluated. For sperm injection, the motile spermatozoon were aspirated by Pasture pipette and then transferred to a 10% PVP droplet. The best morphologically well-shaped spermatozoa were selected for the microinjection procedure. Each spermatozoa was immobilized by touching its tail near the mid-piece with an injecting pipette, and then aspirated from the tail. The injected oocytes were washed twice, then individually placed in fresh droplets of G1 covered with mineral oil.

### Oocyte evaluation

The morphological characteristics of the MII oocytes were evaluated by inverted microscope just prior to microinjection. The characteristics employed for the assessment of oocyte morphology were: a. normal oocytes had clear cytoplasms with homogenous fine granularity; b. granular oocytes, dark with granularity either homogenous in the whole cytoplasm or concentrated in the central portion of the oocyte; c. cytoplasmic inclusions comprised of vacuoles presumed to be of endocytotic origin; d. anomalies of zona pellucida (ZP); e. fragmented polar body; f. non-spherical shaped oocyte; g. wide previtelline space (wPVS); h. refractile bodies (RF); i. bull’s eye; j. debris in the PVS; and k. smooth endoplasmic reticulum cluster (SERc) (8).

### First polar body evaluation

**Fig 1:**
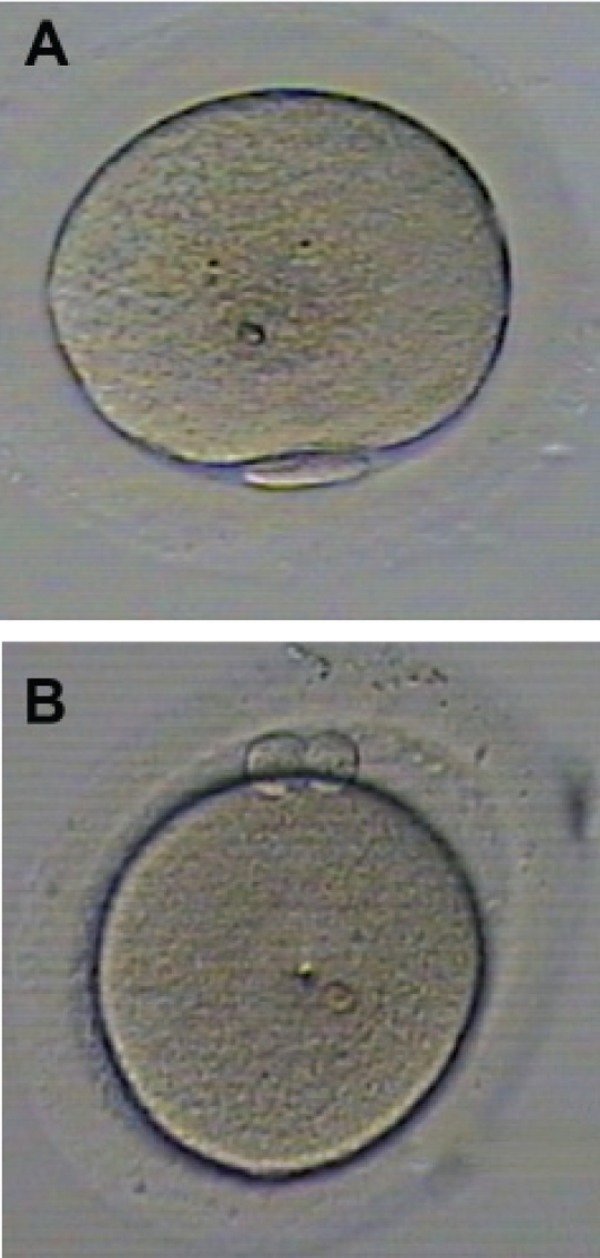
MII oocytes after denudation of cumulus cell before microinjection. a: normal 1^st^ PB and b: fragmented 1^st^ PB.

After denudation, we evaluated all oocytes for 1^st^ PB morphology. The oocytes according to their polar bodies were divided into two groups of A (normal intact 1^st^ PB) and B (abnormal fragmented 1^st^ PB) (Fig 1). Other abnormalities, such as RF, wPVS, central and general granulation, bull’s eye, vacuoles, SERc, debris in the PVS, as well as oocyte shape and color were noted.

### Fertilization evaluation

The injected oocytes were incubated followed by fertilization evaluation 18-19 hours after injection by visualizing the oocytes under a microscope and determining the presence of 2PN.

### Embryo evaluation and transfer

About 48 hours post-injection, we evaluated the embryos according to the procedure of Hill et al. (23). Briefly, grading was as follows: grade A, equal size blastomeres without fragmentation; grade B, slightly unequal blastomeres, up to 10% cytoplasmic fragments; grade C, unequal sized blastomeres up to 50% fragments and large granules; and grade D, unequal blastomeres with significant fragmentation and large black granules. We considered grades A and B to be good quality embryos, whereas grades C and D were poor quality embryos.

### Inclusion and exclusion criteria

All retrieved oocytes were included in the study. No oocytes were cryopreserved or discarded. Egg donation, natural cycles and degenerated oocytes after microinjections due to mechanical error were excluded from the study.

### Statistical analysis

Data was presented as mean ± SE. Statistical analysis chi-square and Fisher`s exact tests were chosen. Data were presented as odds ratio (OR), 95% confidence interval (95% CI) and p value. The ORs referred to fertilization rate and good quality or early cleaved embryos. Independent sample ttests were used wherever appropriate. P<0.05 was considered significant. Statistical analysis was done with the Statistical Program for Social Science (SPSS 16.0, Chicago, IL) software.

## Results

A total of 286 oocytes were normally fertilized, of which 179 were normal and 107 had fragmented 1^st^ PBs. Additionally, 287 embryos were formed of which 179 were good embryos (119 normal oocytes and 60 fragmented 1^st^ PB). From the total of 287 embryos, 213 had early cleavage that resulted from 139 normal oocytes and 74 fragmented 1^st^ PB (Table 1).

**Table 1:** Frequencies in groups A and B


	Normal oocytes(group A)	Fragmented 1^st^ PB oocytes(group B)

**MII oocytes**	291 (61.9%)	179 (38.1%)
**Fertilized oocytes**	179 (62.6%)	107 (37.4%)
**Good quality embryos**	119 (66.5%)	60 (33.5%)
**Early cleavage embryos **	139 (65.2%)	74 (34.8%)


The data showed that 27% of the oocytes had fragmented 1^st^ PB with no other morphological abnormalities, while the remainder were associated with other abnormalities. There were 46.1% of the oocytes that had double defects and 26.9% of the oocytes had multiple defects. In group B, RF (19%) and granulation (9%) were double defects; wPVS with RF (10%) were the most common abnormalities observed for multiple defects. Overall, other abnormalities with fragmentation of 1^st^ PB for both double and multiple defect oocytes was less than 10%. The least anomaly combined with 1^st^ PB fragmentation was darkness in the oocyte cytoplasm (0.6%). For oocytes with multiple defects, the least common anomaly was bull’s eye with debris in the PVS (0.6%).

**Table 2:** Comparison of fertilization and embryo development rates between both groups


	Normal intact 1^st^ PB (group A)	Abnormal fragmented 1^st^ PB (group B)	Odds ratio (95% CI)	p value

**Fertilization rate**	61.5%	59.8%	1.07 (0.73-1.57)	NS
**Good quality embryos **	66.5%	55.6%	1.58 (0.97-2.59)	NS
**Embryo cleavage rate**	77.7%	68.5%	1.59 (0.93-2.73)	NS


CI: confidence interval Chi-square test was used for analysis

**Table 3:** Maternal age comparison for fertilization rates and embryo development


	1^st^ PB morphology		p	Fertilization		p	Embryo quality		p	Cleavage rate		p

**Maternal**	**A**	**B**	**NS**	**yes**	**no**	**NS**	**good**	**poor**	**0.016**	**early**	**late**	**NS**
**age**	32.3 ± 0.3	32.8 ± 0.4		32.3 ± 0.3	32.9 ± 0.4		31.8 ± 0.3	33.3 ± 0.5		32.2 ± 0.3	32.8 ± 0.6	


Independent sample t test was used for analysis

There was no significant relationship between groups A and B in terms of oocyte fertilization rate; 1^st^ PB morphology did not predict fertilization rate. The rates of good embryo formation for groups A was 66.5%, whereas it was 55.6% for group B. Cleavage rates for group A was 77.7% and 68.5% for group B. The embryo development rates were not statistically significant in the two groups (Table 2).

Women with group A had a mean age of 32.3 ± 0.3 years, whereas those with group B were 32.8 ± 0.4 years, which was not statistically significant. However, we found a significant difference between maternal age in groups A and B (p=0.016) with regard to the formation of good embryos (Table 3).

## Discussion

Because of different nuclear maturity, the retrieved oocytes post ovarian hyper-stimulation show different grades of 1^st^ PB morphology. The presence of 1^st^ PB and its observation by an embryologist before ICSI is very important because extrusion of the 1^st^ PB reflects MII oocyte maturity. One of the attractive issues in ART is finding criteria(s) to predict which oocytes will fertilize and which oocyte characteristic(s) may affect embryo development during the ART procedure.

Our results demonstrate no correlation between 1^st^ PB morphology and fertilization rate, embryo quality or even cleavage rate in women undergoing ICSI treatments. There is no significant difference between maternal age in groups A and B. Also, patients’ ages were not related to fertilization and cleavage rates, similar to the findings of Ciotti et al. (16) but is related to embryo quality. In the literature, the prognostic value of 1^st^ PB morphology for fertilization rate, embryo quality and cleavage rate is controversial. Our findings are similar to previous studies reported by reproductive scientists (11, 14-17, 19, 24, 25), but conflict with reports from others (9, 10, 13). One reason for this contradiction may be related to the methodological variation in oocyte evaluation. Xia (9) and Mikkelsen and Collouge (24) have reported that the 1^st^ PB morphology combined with perivitelline space and cytoplasmic inclusions can be used as prognostic factors for fertilization rate and cleavaged embryo quality (9, 24), however we only evaluated 1^st^ PB morphology. The other reason for this discrepancy may be related to the use of a different 1^st^ PB grading system. Some studies divide 1^st^ PB morphology simply into two groups of normal and fragmented, as we did. However, others may grade 1^st^ PB according to criteria such as surface, size and maturity (9, 10, 13).

Considering the positive relationship between 1^st^ PB morphology and time elapsed in culture, therefore, PB morphology may alter after a few hours, and it can change according to the timing of the observation. Ciotti et al. have noted that 1^st^ PB fragmentation is related to the time elapse between retrieval, denudation and ICSI performance (16). They checked a subgroup of oocytes twice (at the moment of denudation and injection, respectively) for 1^st^ PB fragmentation and have observed different degrees of fragmentation at the first and second efforts (11.1% and 22.8%, respectively). However, as they increased the time elapsed for denudation to >3.5 hours, the fragmentation rate of the oocytes was 26.7%. Another dispensary may be related to the procedure that occurs for the time of the 1^st^ PB evaluation and performing the ICSI. In different studies, distinct ICSI procedures were applied, but timing for cumulus cell denudation and ultimately the time in which the 1^st^ PB was evaluated may not be the same. It is well known that, because of controlled ovarian hyperstimulation, not all retrieved oocytes have the same quality, and different ovarian hyperstimulation protocols may be applied in studies. Therefore, oocyte quality may be influenced by the aforementioned protocols.

Moreover, Verlinsky and his colleagues in 2003 have shown that PB morphology is not related to the genotype analyzed for aneuploidy in patients who underwent preimplantation genetic diagnosis (PGD). They noticed no correlation between polar body shape and genetic constitution of the oocyte (19). In addition, they detected 1^st^ PB morphology grading changes in terms of fragmentation in over one-third of the oocytes studied. Hence, 1^st^ PB morphology assessment may not serve as a reliable marker for assessment of oocyte quality and competence.

## Conclusion

The data demonstrated that 1^st^ PB morphology does not appear to be a prognostic factor for oocyte competence in the process of fertilization and early embryo development in ICSI cycles.
